# Moving the needle for COVID-19 vaccinations in Nigeria through leadership, accountability, and transparency

**DOI:** 10.3389/fpubh.2023.1199481

**Published:** 2023-09-21

**Authors:** Toluwanimi Ojeniyi, Amenze Eguavoen, Fejiro Chinye-Nwoko

**Affiliations:** ^1^Programs Department, Nigeria Solidarity Support Fund, Lagos, Nigeria; ^2^Management, Nigeria Solidarity Support Fund, Lagos, Nigeria

**Keywords:** COVID-19 vaccination, leadership and accountability, parallel funding, vaccine acceptance, vaccine uptake

## Abstract

**Background:**

The first set of vaccines arrived in Nigeria in March 2021. The National Primary Health Care Development Agency (NPHCDA) set out to vaccinate at least 70% percent of Nigeria’s eligible population, i.e., 111,776,503 people, by December 2022. As of June 2021, only 3% had received at least one dose of the vaccine. This presented a threat to the achievement of NPHCDA’s goal. Nigeria Solidarity Support Fund (NSSF) went into a partnership with NPHCDA to accelerate the uptake of COVID-19 vaccinations across Nigeria over 3 months.

**Methods:**

Across Nigeria’s 6 geopolitical zones, 6 states were selected, namely: Adamawa, Edo, Imo, Katsina, Nasarawa, and Ogun states based on performance, political will, and absence of external resources. A two-pronged approach was implemented: unrestricted funding to the sub-national level and providing technical support at the national level.

**Results:**

5 out of 6 states received unrestricted funding to ramp up vaccination coverage. They also received adequate vaccine supplies. A total of 12,000 healthcare workers were trained on safe immunization practices and multiple communities were engaged across the 133 local government areas (LGAs) through religious and community leaders. After 6 months, there was an average of 35% increase in the uptake of COVID-19 vaccines in the 5 states. An indicator tracker was developed for weekly reviews at the national level and the total population vaccinated in Nigeria increased from 6,186,647 to 11,985,336 at the end of the partnership.

**Conclusion:**

Unrestricted funding, though not without its risks, can yield a significant impact on health. The intervention was co-designed with stakeholders and had leadership buy-in, accountability mechanisms, with unrestricted funding. These techniques produced an increase in the vaccination rates in the 5 states and across the country. These elements should be explored for application to other program designs such as routine immunization.

## Introduction

The COVID-19 pandemic presented a serious threat to global public health. The impact of the pandemic was overwhelming, even for developed nations whose already strong health systems were expected to withstand the impact. Predictably, the situation elicited collective social and scientific responses from individuals and governments worldwide; this included the development of a vaccine to protect against the virus (1).

In Nigeria, the COVID-19 pandemic has had a significant impact, both in the number of confirmed cases and the economic and social effects. As of January 2023, Nigeria had reported over 266,463 confirmed cases of COVID-19 and 3,155 deaths (2). At the height of the global outbreak in 2020, the Nigerian government implemented measures such as lockdowns and travel restrictions to slow the spread of the virus. As with many other countries, these measures harmed the economy, with many businesses shutting down and unemployment rising. The Nigerian government was criticized for its handling of the pandemic, with allegations of mismanagement and lack of transparency.

The COVID-19 vaccination campaign is an ongoing effort to vaccinate eligible populations against COVID-19 worldwide, with the aim of achieving herd immunity from the virus. However, due to the changing nature of the virus, this goal was adjusted to vaccinate enough people against COVID-19, rather than to achieve herd immunity, as has been attained with many other diseases (3). Vaccination is expected to ensure protection from the disease, control the rate of infection, and reduce severe outcomes if infection occurs at all. Nigeria received the first set of COVID-19 vaccines in March 2021, through COVAX, the vaccine arm of the Access to COVID-19 Tools (ACT) (4). Through this facility, vaccines were secured from multiple manufacturers and distributed to participating countries based on their needs (5). These vaccines were handed over to the National Primary Healthcare Development Agency (NPHCDA) to coordinate the immunization program across the country. NPHCDA is the arm of the government charged with improving the effectiveness and efficiency of primary healthcare delivery in Nigeria (6), which involves vaccinations. Upon receipt of the COVID-19 vaccines, the NPHCDA set out to vaccinate at least 70 percent of the total 111,776,503 people who were eligible to receive the vaccine by December 2022 (7). The NPHCDA intended to follow a plan of vaccinating 40 percent of the population by December 2021 and the other 30 percent in 2022 (8). Vaccinations kicked off fully in March 2021, with a campaign to raise awareness about the vaccine and keep people informed about where they could get vaccinated. Notwithstanding these collective efforts, the uptake of vaccines remained marginally low and at that rate, there was a real threat against the achievement of NPHCDA’s target for the year 2021 (9). As of July 2021, only 3,441,146 doses of the vaccine had been administered, which was barely 3% of the target population.

While there was a lot of optimism about the COVID-19 vaccination campaign globally, it was anticipated that implementation would not be without challenges. In Nigeria, the low uptake was attributed to funding gaps, which hindered last-mile delivery of vaccines, and challenges with accountability and transparency. After a series of consultative meetings, Nigeria Solidarity Support Fund (NSSF) and the National Primary Health Care Development Agency (NPHCDA) entered a partnership to augment the COVID-19 vaccination campaign across the country. This partnership kicked off in September 2021 (10, 11).

### Objective

This report outlines the strategies employed to scale up COVID-19 vaccination in 5 low performing states in Nigeria.

## Methods

Consultative meetings between NSSF and the NPHCDA revealed that the issues encountered with the COVID-19 vaccine coverage were on two broad fronts; limited funding for program activities at the subnational (state) level and some coordination issues at the national level. Hence, it was imperative that NSSF provided support at both levels to make the desired impact. Therefore, a two-pronged approach was applied through the provision of unrestricted funding at the subnational level and technical support for coordination and monitoring at the national level.

Across the six (6) geo-political zones in Nigeria, six (6) states were selected, namely Edo, Ogun, Nasarawa, Adamawa, Katsina, and Imo state. They were selected based on their status of being the lowest performing states at the time, in each of the geo-political zones. This came to a total of 133 local government areas (LGAs) and 513 implementing wards across the states. One of the six selected states, however, did not successfully implement the project.

The partnership kicked off in October 2021 and ran for a period of 3 months; which ended in January 2022.

### Advocacy for last mile delivery of vaccines and demand creation

The COVAX facility helped to ensure the continued supply of vaccines to Nigeria. However, at the initial stage, the supply of vaccines was sub-optimal, making the quantity of vaccines available insufficient to meet the demand. Hence, only a few facilities in each of the states had received vaccines as of September 2021, when the partnership kicked off. NSSF worked with the NPHCDA to advocate for an increase in the supply of vaccines and to ensure that the vaccines were sufficiently distributed to the local level. This was done over a 4-week period, between October and November, 2021, to establish a reliable chain of supply and ensure continuity.

Alongside advocacy for vaccine supply, health education efforts were doubled in the participating states. This component was essential for increasing awareness about the vaccines and where people could get them, to drive demand. The primary healthcare development teams in each state worked with their media partners to develop additional information, education, and communication (IEC) materials, including jingles. The jingles were aired on radio and television. IEC materials were developed and deployed as an ongoing activity from October to December 2021.

In addition to the jingles, call-in programs were aired on the radio, which allowed people to ask questions about COVID-19, the vaccine, and its effects.

Road shows and rallies were conducted in all 5 states, to reach communities and settlements where other methods of communication may not have reached. These took place within the first two weeks of October 2021, following the flag-off events.

### Training of healthcare workers

Important to any immunization exercise or campaign is the constant availability of the vaccines, as well as the mechanisms for last-mile delivery, reverse logistics, and waste disposal. Doctors, nurses, midwives, laboratory technicians, pharmacists, community health officers (CHO), and community health extension workers (CHEW) participated in a three-day intensive training, which was conducted from October 3rd–5th, 2021 in each of the LGAs and their respective communities. In addition, the NPHCDA deployed Community Health Influencers, Promoters, and Supporters (CHIPS) to drive uptake of the vaccines.

All healthcare workers and community leaders were trained to follow these steps:

T: Traditional method of vaccinating target populations using desk review of available data sources, identifying the vaccination sites, and rolling out.
E: Electronic self-registration for health workers and the public; a link that provides an online form was provided.
A: Assisted electronic registration.
C: Concomitant e-registration during walk-ins to fixed sites/health facilities.
H: House-to-House registration using volunteers for additional push to rapidly increase the e-registration.

This is the TEACH strategy. In addition, the Electronic Management of Immunization Data (EMID) application was launched by the Federal Ministry of Health, to capture and store information on COVID-19 vaccination activities. The healthcare workers were also trained to use the application and ensure that data was captured to reflect the actual situation.

Information gaps among the healthcare workers were addressed, to increase their confidence in the efficacy of the vaccine. Ensuring that the healthcare workers were confident in the vaccine was essential for promoting the acceptance of the vaccine by beneficiaries in the various communities.

### Data management and transparency

At the national level, indicators were developed to ensure accountability and uniform reporting across board. Under the direction of the State Primary Healthcare Development Agency (SPHCDA) in each state, monitoring and evaluation (M&E) teams were set up at the local government and state levels. The M&E teams and their associated M&E focal persons were trained in the use of the TEACH strategy and the Electronic Management of Immunization Data (EMID) application.

Data management was a key part of the campaign from its initiation in September 2021; this continued through the period of the partnership and afterwards, until October 2022. Immunization data, which was validated by *ad-hoc* staff, was reported manually and *via* the EMID. The records were revalidated each week to identify and reconcile discrepancies between data called-in and EMID records. These assessments were corroborated by spot checks with the LGA focal persons. Disaggregated data was reviewed to identify bottlenecks in team performance, which also informed remuneration.

### Campaign coordination and technical support

Under the guidance of the NPHCDA, the states conducted weekly data review meetings between October 2021 and January 2022. These meetings provided an avenue to go over the data reported for the LGAs in each state. During the meetings, the strategies being implemented were also evaluated and ineffective strategies were revised.

**Figure 1:**
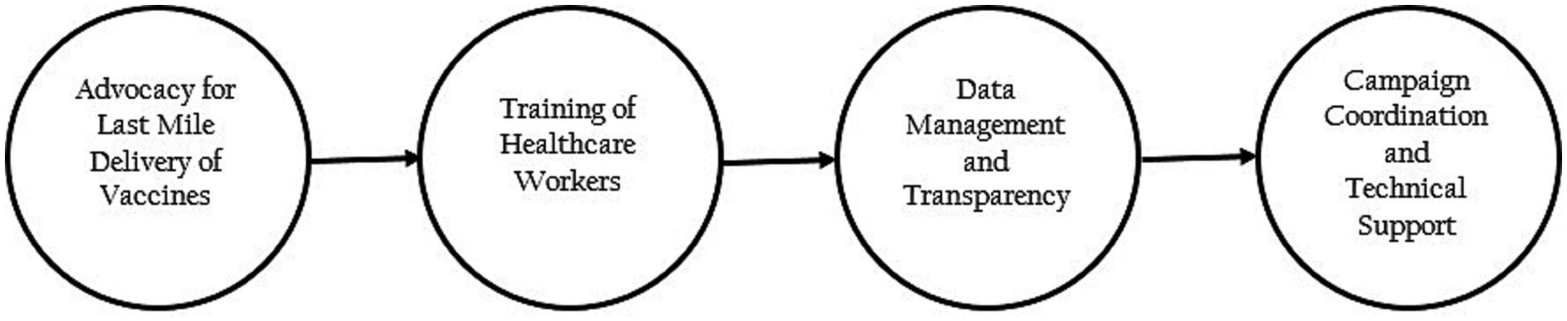
The framework used to increase COVID-19 vaccine uptake across Nigeria (Nigeria, 2022).

At the national level, review meetings were conducted monthly, and the states were ranked based on their performance. Based on the results of the rankings, low-performing states were provided additional support centered on state-specific needs (Figure 1).

## Results

At the time that the partnership began, 1,226,311 eligible people in Adamawa had received the COVID-19 vaccine. This figure increased by 175,336 by December 2021 and an additional 93,478 at the end of January 2022. The support from NSSF contributed to a 17.98% (268,814) increase in the vaccination uptake, resulting in the vaccination of 1,495,125 people in Adamawa at the end of the campaign.

Similarly, in Imo state, 2,940,851 people were targeted for the COVID-19 vaccination. During the implementation period, a total of 90,501 people received the first dose of the vaccine, while 53,190 people received the second dose.

Katsina state, which is the largest of the six states, had 5,345,789 people targeted for vaccinations. When the campaign in the state ended, 2,034,094 people had received at least one dose of the COVID-19 vaccination. This was achieved, despite the insecurity challenges that made 17 LGAs inaccessible to the vaccination team.

Before receiving support from NSSF, 1,306,185 people in Nasarawa had received at least one dose of the COVID-19 vaccine. During the period of NSSF’s partnership with the NPHCDA, there was a 73.17% (955,768) increase in the vaccination uptake in Nasarawa, resulting in the vaccination of 2,261,953 people at the end of the campaign.

In Ogun state, 112,167 people were vaccinated during the period. This contributed to the total of 414,221 people who had received at least one dose of the COVID-19 vaccine in Ogun state, which is 20.71% of the target population of 2,000,000 people.

In Edo state, the grant was not implemented due to some bureaucratic issues at the state level. The issues were resolved eventually, but the implementation period had elapsed, therefore, results from the state were not included in the data reported.

At the end of the implementation period in the five states, COVID-19 vaccination coverage increased across board. At least 12,000,000 people were reached through the vaccine advocacy campaigns and 12,000 healthcare workers benefited from the training programs on safe immunization practices. Within 3 months, the population vaccinated in the 5 states was 3,514,534 people and there was a steady increase in the uptake of the vaccines across the states. As of March 2022, 5,235,493 people had been vaccinated in the five states, which is almost half of the 11,985,336 people who had been vaccinated in Nigeria at the time.

**Figure 2:**
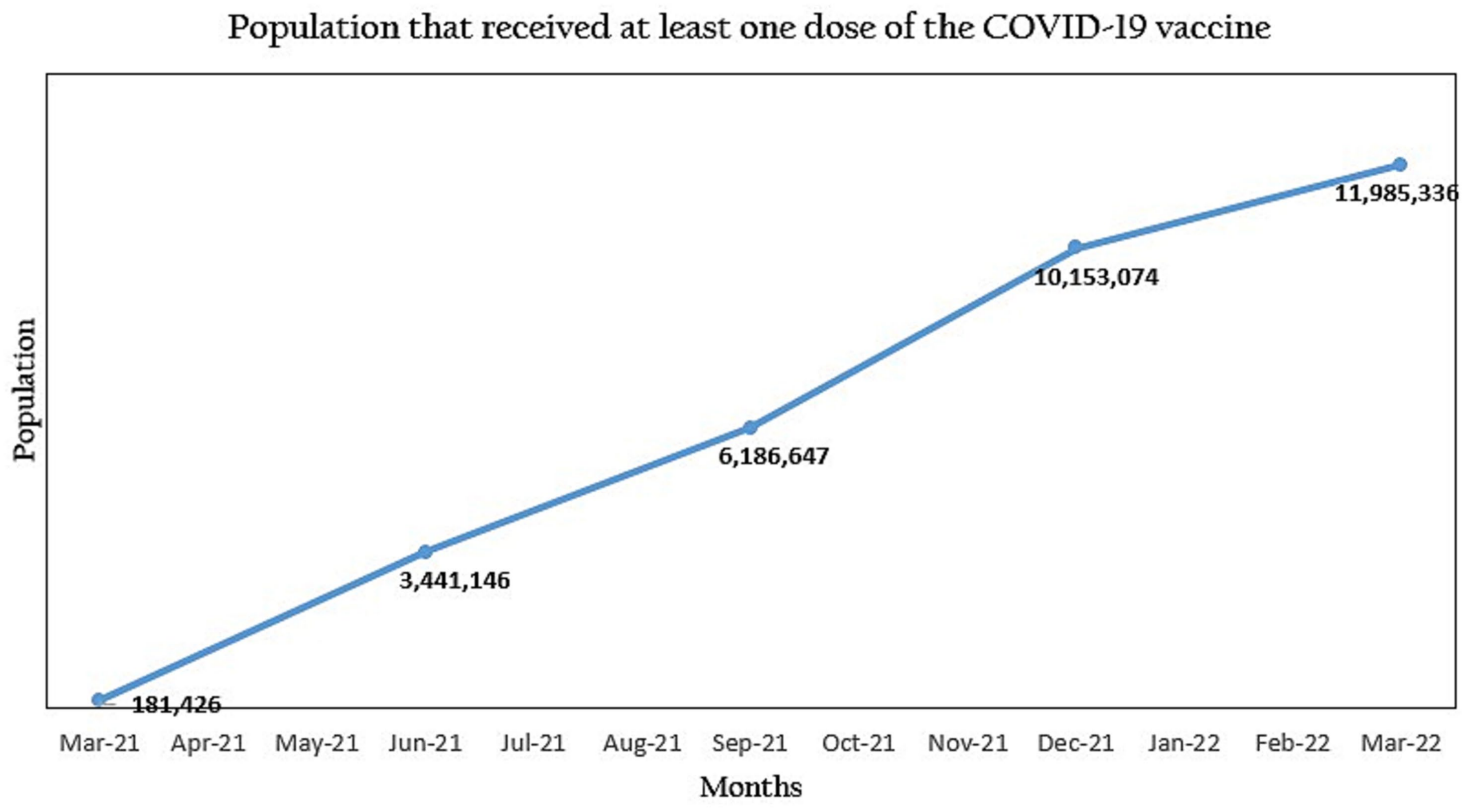
Progression of the COVID-19 vaccine uptake in Nigeria during the National Primary Healthcare Development Agency (NPHCDA) and Nigeria Solidarity Support Fund (NSSF) partnership.

In addition, owing to the support provided by NSSF, Nasarawa state and Ogun state were reported to be the first and ninth positions, respectively, in the national COVID-19 vaccination coverage report as of July 20th, 2022 (Table 1; Figure 2).

**Table 1:** Progression of COVID Vaccinations in the 5 states between October 2021 and March 2022.

State	Oct-21	Nov-21	Dec-21	Jan-22	Feb-22	Mar-22
Adamawa	147,096	170,692	245,529	356,056	465,616	627,107
Imo	119,803	134,163	160,020	188,100	205,514	250,989
Katsina	205,165	222,031	384,310	497,399	596,504	729,147
Nasarawa	174,838	237,998	522,094	1,128,628	1,756,642	2,116,571
Ogun	456,294	619.652	921,309	1,139,049	1,441,437	1,511,679

## Discussion

In Nigeria, lessons were taken from the experience that has been gained through the years in other immunization campaigns like the polio eradication campaign. Nasarawa state, which eventually got applauded for the increased coverage in the state, reported that they piggybacked on the Reach Every Ward (REW) strategy and were supported by the CHIPS. The importance of community ownership was also underscored, through the leaders who were educated about the vaccine and subsequently vaccinated, as well as the CHIPS agents, who were representatives of their communities and could be trusted to give relatable advice.

Advocacy plays a very important role in determining the uptake of vaccines in any region. While advocacy can often be seen as a one-way train, it is most effective when communication is bidirectional therefore, it is important to understand the source of misinformation or mistrust when fashioning advocacy messages. Nigerians are generally religious people; hence they tend to get and trust information from their religious and traditional leaders, over healthcare workers and ministries. Therefore, educating these leaders was very important and beneficial for driving vaccination uptake in many areas. Some countries, like Zimbabwe, even went as far as allowing only vaccinated worshipers to attend gatherings physically and this further encouraged people to get vaccinated (12). In addition, a lot of work was done to correct misinformation and disinformation by employing champions within all communities, like the CHIPS agents, to strengthen communication. This was further supported by rallies and road shows, which were held every week. During advocacy visits, people were encouraged to get information about COVID-19 and the vaccine from confirmed sources, like the NCDC and NPHCDA websites, which aligns with what was done in Uganda and Tanzania (13).

Alongside healthcare workers, social mobilizers who were non- healthcare workers that lived within communities and were willing to be educated, played a very important role in creating demand for COVID-19 by educating other community members on the vaccine and its efficacy (10). It was upon this strategy that the CHIPS thrived and made an impact, creating and sustaining demand for vaccines in Nasarawa and Adamawa.

Data management was a very important part of the campaign and constituted a large part of the training conducted for healthcare workers. Electronic management of data was initially frowned upon by many countries in sub-Saharan Africa especially, as it was considered ‘elitist’ and not suited to the rural areas and people who live there. However, this was resolved in Nigeria using mobile vaccination teams that were tasked with going into those communities and registering the beneficiaries, then administering vaccines to them. A similar process of data management was employed in Rwanda, where the DHIS2 was used for registration of COVID-19 vaccine beneficiaries and automation of reminders (11). This process ensured a quick transmission of COVID-19 vaccination data to the national level; insights from which were used to make decisions about the strategies to be employed for increased demand creation.

While this partnership followed some methods that are common in vaccination campaigns, the success recorded may not have been achieved without the principle of additionality. NSSF was intent on working with the NPHCDA to contribute to the global goal of reducing COVID-19 infection rates through vaccinations. The grant was not implemented as a parallel project by NSSF or another private institution but the organization consulted with the NPHCDA, got information about the gaps that needed to be addressed, and worked with them to mitigate the challenges. This process allowed the NPHCDA and NSSF to geometrically increase their reach and impact.

The participating states were granted unrestricted funding for their activities. Funding granted was not tied to grant lines and this allowed flexibility in implementation and implementation research on the go. The federal and state teams owned the project and set population-based targets for each state. Hence the state teams were driven to work hard at achieving their targets. The state teams were able to take context-specific approaches to address their challenges and improve the processes as they saw fit.

## Conclusion

This intervention, which was designed for 6 of the 36 states in Nigeria, but implemented in only five states was implemented through the partnership between a private sector entity, NSSF, and the government (NPHCDA). While the implementation was largely the responsibility of the NPHCDA, it was evident that immunization targets can be achieved and surpassed when stakeholders, including beneficiaries of the immunization programs, work together.

Also, the intervention showed that despite the risks that come with unrestricted funding, grants provided through this approach can yield a significant impact on health. Although Nigeria still has some work to do to achieve herd immunity, leadership buy-in, accountability mechanisms, and unrestricted funding are important factors that can significantly promote vaccination campaigns and move the needle. This was evident in the 5 states that moved from being among the lowest performing states in the country to the best in terms of the COVID-19 vaccination campaign. Their performance motivated other states to source additional funding for last-mile delivery.

While success was recorded with the COVID-19 vaccination campaign in the 5 states, a lot of work remained undone due to limited funding. This further proves that financial and technical support from donor organizations and the private sector is still necessary to catalyze the government’s efforts and achievements in vaccine coverage and other health interventions.

## Recommendation

The grant was not implemented as a parallel project, rather it was implemented in the principle of additionality. This allowed the states to scale impact while taking ownership of the projects and applying context-specific methods to vaccinate the populations in each state. Applying this to other grants will allow flexibility of the program and produce the expected impact.

## Limitation

This paper was developed based on a grant that was awarded to five states in Nigeria for a three-month period. NSSF did not provide support for longer than 3 months due to competing program needs. However, the lessons learned from this intervention can be replicated with grants to be awarded in Nigeria and other LMICs.

## Data availability statement

The original contributions presented in the study are included in the article/supplementary material, further inquiries can be directed to the corresponding author.

## Author contributions

All authors listed have made a substantial, direct, and intellectual contribution to the work and approved it for publication.

## Conflict of interest

The authors declare that the research was conducted in the absence of any commercial or financial relationships that could be construed as a potential conflict of interest.

## Publisher’s note

All claims expressed in this article are solely those of the authors and do not necessarily represent those of their affiliated organizations, or those of the publisher, the editors and the reviewers. Any product that may be evaluated in this article, or claim that may be made by its manufacturer, is not guaranteed or endorsed by the publisher.
